# Mapping population access to essential surgical care in Liberia using equipment, personnel, and bellwether capability standards

**DOI:** 10.1093/bjs/znac377

**Published:** 2022-12-05

**Authors:** Håvard A Adde, Alex J van Duinen, Benetta C Andrews, Juul Bakker, Kezelebah S Goyah, Øyvind Salvesen, Swaliho Sheriff, Terseer Utam, Clarence Yaskey, Thomas G Weiser, Håkon A Bolkan

**Affiliations:** Department of Clinical and Molecular Medicine, Faculty of Medicine and Health Sciences, NTNU—Norwegian University of Science and Technology, Trondheim, Norway; Department of Clinical and Molecular Medicine, Faculty of Medicine and Health Sciences, NTNU—Norwegian University of Science and Technology, Trondheim, Norway; Department of Surgery, St Olav’s Hospital, Trondheim University Hospital, Trondheim, Norway; Liberia College of Physicians and Surgeons, Monrovia, Liberia; Department of Public Health and Nursing, Faculty of Medicine and Health Sciences, NTNU—Norwegian University of Science and Technology, Trondheim, Norway; Lifebox Foundation, Monrovia, Liberia; F. J. Grante Memorial Hospital, Greenville, Liberia; Department of Public Health and Nursing, Faculty of Medicine and Health Sciences, NTNU—Norwegian University of Science and Technology, Trondheim, Norway; Lifebox Foundation, Monrovia, Liberia; Department of Surgery, Liberia Governmental Hospital, Tubmanburg, Liberia; Lifebox Foundation, Monrovia, Liberia; Department of Surgery and Traumatology, Redemption Hospital, Monrovia, Liberia; Lifebox Foundation, Monrovia, Liberia; Department of Surgery, Stanford University, Stanford, California, USA; Department of Surgery, Stanford-Surgery Policy Improvement Research and Education Center, Stanford University, Palo Alto, California, USA; Department of Clinical Surgery, University of Edinburgh, Edinburgh, UK; Lifebox Foundation, London, UK; Department of Surgery, St Olav’s Hospital, Trondheim University Hospital, Trondheim, Norway; Department of Public Health and Nursing, Faculty of Medicine and Health Sciences, NTNU—Norwegian University of Science and Technology, Trondheim, Norway

## Abstract

**Background:**

Accurate surveillance of population access to essential surgery is key for strategic healthcare planning. This study aimed to estimate population access to surgical facilities meeting standards for safe surgery equipment, specialized surgical personnel, and bellwether capability, cesarean delivery, emergency laparotomy, and long-bone fracture fixation and to evaluate the validity of using these standards to describe the full breadth of essential surgical care needs in Liberia.

**Method:**

An observational study of surgical facilities was conducted in Liberia between 20 September and 8 November 2018. Facility data were combined with geospatial data and analysed in an online visualization platform.

**Results:**

Data were collected from 51 of 52 surgical facilities. Nationally, 52.9 per cent of the population (2 392 000 of 4 525 000 people) had 2-h access to their closest surgical facility, whereas 41.1 per cent (1 858 000 people) and 48.6 per cent (2 199 000 people) had 2-h access to a facility meeting the personnel and equipment standards respectively. Six facilities performed all bellwether procedures; 38.7 per cent of the population (1 751 000 people) had 2-h access to one of these facilities. Bellwether-capable facilities were more likely to perform other essential surgical procedures (OR 3.13, 95 per cent c.i. 1.28 to 7.65; *P* = 0.012). These facilities delivered a median of 13.0 (i.q.r. 11.3–16.5) additional essential procedures.

**Conclusion:**

Population access to essential surgery is limited in Liberia; strategies to reduce travel times ought to be part of healthcare policy. Policymakers should also be aware that bellwether capability might not be a valid proxy for the full breadth of essential surgical care in low-income settings.

## Teaser

This observational study, which mapped population access to essential surgery in Liberia, found that approximately 60 per cent of the population cannot reach a bellwether-capable facility within 2 h of travel. Furthermore, these facilities did not deliver the full breadth of essential surgical care. Long travel time is a major challenge for many patients trying to reach surgical care in Liberia; the ones who do arrive in time might receive inadequate care owing to poor availability of essential surgical services.

## Introduction

Although remarkable progress has been made over past decades to advance good health and well-being for people worldwide, significant disparities in population access to essential surgical services remain^1^. First-level hospitals are particularly cost-effective for the delivery of essential surgical care, and these facilities should be widely dispersed geographically to make them easily accessible^2^. Geographical access to essential surgical services was emphasized as a key metric by the Lancet Commission on Global Surgery^1^, targeting that 80 per cent of a country’s population should be able to reach a facility performing the bellwether procedures (caesarean section, laparotomy, and open fracture treatment) within 2 h of travel.

Access to essential surgical care depends on both geographic accessibility and availability of necessary resources to perform operations^1^. The bellwether procedures have gained a central role in global surgery benchmarking owing to their association with a wider range of other essential procedures^3^. A geospatial analysis of population access to surgical services in Ghana found that ability to perform the bellwether procedures was a useful proxy for essential surgery more broadly^4^. However, other studies have rather used equipment and personnel standards to report population access to essential surgical services^5,6^, or expanding surveillance to include a selection of representative operations^7^. There is a need to better understand surgical standards and their relevance as proxies for the full breadth of essential surgery.

**Fig. 1:**
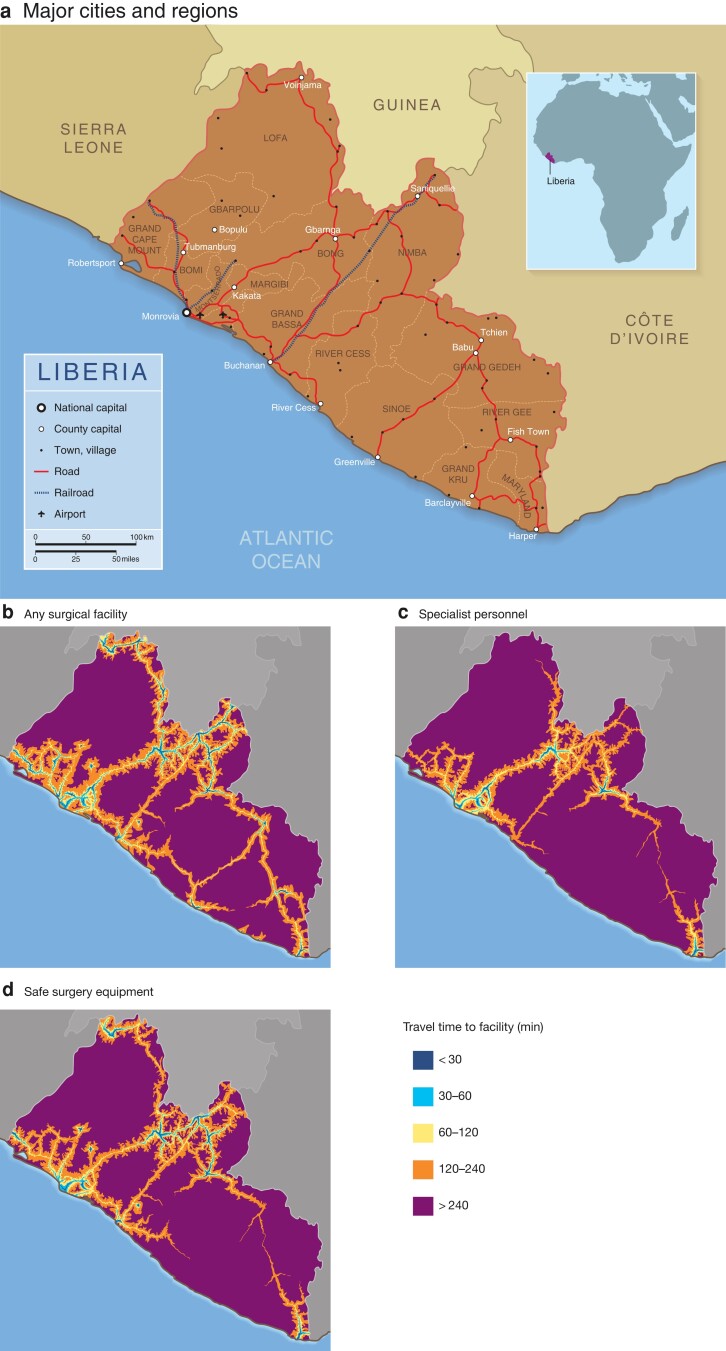
Major cities and regions of Liberia, and surgical facilities Maps showing **a** major cities and regions, **b** population 2-h access to any surgical facility, **c** facilities with both a surgeon and an obstetrician/gynaecologist specialist present, and **d** facilities with safe surgery equipment available.

Liberia is a small West African nation (*Fig. 1a*) with a health system heavily affected by a brutal civil war and recent Ebola virus outbreak^8,9^. The country has a low-income economy^10^, and is ranked at the lower end of the United Nations Human Development Index^11^. A recent nationwide enumeration of operative numbers and surgical providers described a surgical volume of 462 operations per 100 000 population^12^ and a surgical specialist density of 1.6 per 100 000 population^13^, which is far below the Lancet Commission on Global Surgery targets^1^. Previous estimates have suggested that approximately 39 per cent of Liberians live more than 2 h away from the nearest emergency hospital^14^. However, many hospitals do not meet basic surgical standards, which affects population access to surgical services^6^.

This study aimed to estimate population access to surgical facilities meeting standards for safe surgery equipment, specialized surgical personnel, and bellwether capability, and evaluate the validity of using these standards to describe the full breadth of essential surgical care needs in Liberia.

## Methods

### Ethical consideration

The data collection for this study was approved by the Institution Review Board, University of Liberia. The Regional Committee for Medical and Health Research Ethics in central Norway exempted this study from review (number 2018/1008). All administrative leaders consented on behalf of their facility to participate in the study.

### Data collection

A nationwide observational study of surgical facilities was undertaken in Liberia in 2018. All healthcare facilities performing surgical procedures requiring general, regional, or local anaesthesia in an operating theatre between September 2017 and August 2018 were eligible for inclusion. Facilities were visited between 20 September and 8 November 2018, and data were collected using the Lancet Commission on Global Surgery Hospital Assessment Tool and through review of operative logs. A sample of 4 months preselected from the logbooks, representing all seasons (October, January, April, July), were transcribed into a Microsoft^®^ Excel data set (Microsoft, Redmond, Washington, USA). An online application was used to record global positioning system coordinates during facility visits. An in-depth description of the data collection has been reported previously^12^.

### Definitions and outcome measures

The ability to deliver essential surgical care was considered using three dimensions of surgical standards: availability of safe surgery equipment; presence of specialized surgical personnel; and provision of the bellwether procedures.

Eight equipment items were considered (availability of pulse oximeter, adult bag mask, oxygen, suction, intravenous fluids, sterile gloves, skin preparation solution, and sterilizer)^5^, and these needed to be readily available for the facility to be described as a safe surgery facility.

Facilities were categorized as specialist personnel-capable if they had both a specialist surgeon and a specialist obstetrician/gynaecologist present; their presence was validated using operative logs.

All facilities with operative logs documenting the performance of caesarean section, laparotomy, and open fracture treatment (so-called bellwether procedures) were defined as bellwether-capable^1^. Treatment of open fracture was defined as any care of an open fracture including fracture reduction and/or external fixation and/or traction reported in the operating theatre logbooks^3^. Facilities reporting open reduction and internal fixation of fractures were also included as open fracture treatment-capable. Any surgical procedure listed in the WHO Situation Analysis Tool^3^ and/or listed as especially cost-effective in the third edition of the Disease Control Priorities^2^ was defined as essential. The term ‘other essential procedures’ points to all essential procedures except the bellwethers themselves. Population poverty levels were defined according to the 2016 Liberian Household Income and Expenditure Survey^15^.

### Geospatial modelling

A geospatial model was built using the open source WHO analysis tool AccessMod 5.6.0^16^ and the Geographical Information System software QGIS (version 3.16; Open Source Geospatial Foundation Project, Grüt (Gossau), Switzerland). Input data layers consisted of a digital elevation model with a resolution of 94 m and land cover data from the 2016 Africa land cover (contains modified Copernicus data (2015/2016), European Space Agency Climate Change Initiative–Land Cover project 2017), road and hydrographic networks from OpenStreetMap^17^, population density data from the WorldPop database^18^, and geocoded surgical facility locations.

Roads were classified as primary (including trunk roads), secondary or tertiary (including unclassified roads), and a travel speed scenario was created for the road network. Recent studies have found that widely used travel scenarios underestimated self-reported travel times both in Rwanda^19^ and Sierra Leone^20^. The study from Sierra Leone, which is a neighbouring country to Liberia, found that more conservative travel speeds correlated better with reported travel times^20^. Conservative travel speeds^21^, which were also validated by local members of the study team, were therefore applied in the present geospatial model.

The accessibility analysis was run in AccessMod to estimate population travel time to the closest surgical facility meeting the defined surgical standards. The accessibility module was also used to identify five facilities with the greatest potential impact on population 2-h access should their bellwether capability be scaled up. The module was run individually 45 times including the 6 bellwether-capable facilities and 1 of the 45 non-capable facilities. The facilities with the greatest impact were then analysed in combination to adjust for potential overlaps in catchment population, which ultimately identified a combination of five facilities most suitable for upscaling.

### Statistical analysis

A mixed binominal regression model with lme4^22^ was built in RStudio version 1.3.1093 (RStudio, Boston, USA) to investigate the association between facility standards and performance of essential surgical procedures other than the bellwethers. These procedures were identified in the facility operating theatre logbooks and counted. In the regression model, facility identification was included as a random effect, whereas the other variables were fixed effects. The effect measure was an OR; an OR of 2 implied a two-fold increased odds of performing any of the other essential procedures compared with the reference. In each facility, the number of surgical providers and the availability of basic surgical infrastructure were summarized. The infrastructure score applied has been described in detail elsewhere^12^. A multivariable model was run adjusting for the total number of surgical providers and infrastructure score at the facility.

## Results

### Population access to facilities meeting surgical standards

In 2018, 52 healthcare facilities reported surgical activity in Liberia, and 51 of these shared surgical data. Including all 51 facilities in the geospatial analysis, 52.9 per cent of the population (2 392 000 of 4 525 000 people) lived within 2 h of travel to 1 of these facilities (*Fig. 1b*).

Limiting the analysis to include only the 27 facilities meeting the safe surgery equipment standard, and the 10 facilities meeting the specialized personnel standard, population access declined; 48.6 per cent (2 199 000 people) and 41.1 per cent (1 858 000 people) of the population could then reach 1 of these facilities within 2 h of travel respectively (*Fig. 1c,d*).

**Fig. 2:**
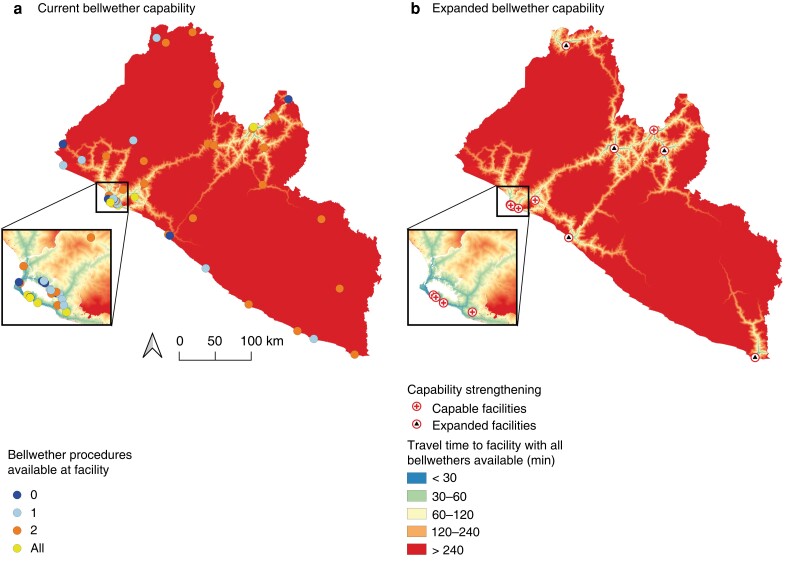
Current and expanded bellwether capability in Liberia **a** Population 2-h access to facilities currently performing the bellwether procedures and **b** an expanded scenario in which five additional facilities are scaled up to full bellwether capability.

**Fig. 3:**
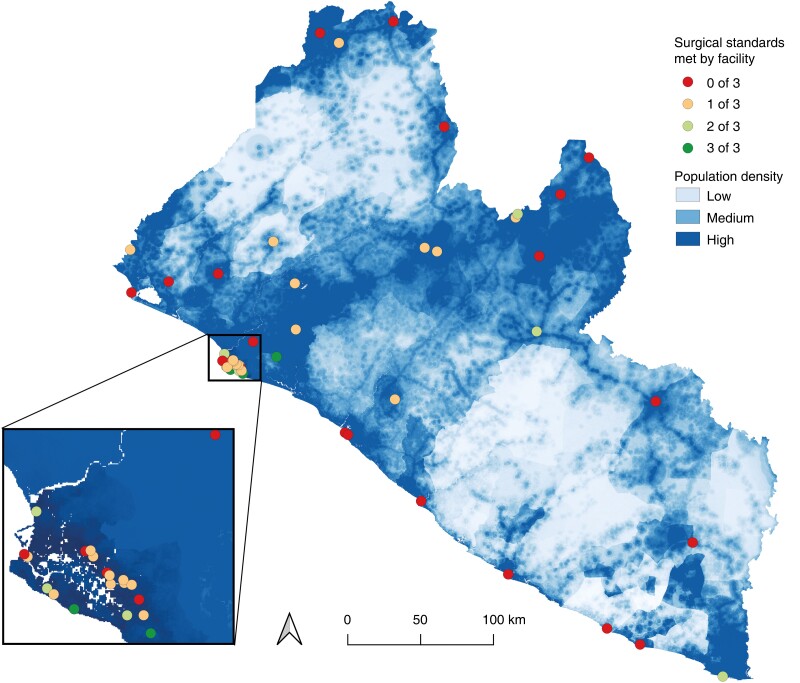
Surgical standards (equipment, personnel, and bellwether capability) in relation to population density

**Fig. 4:**
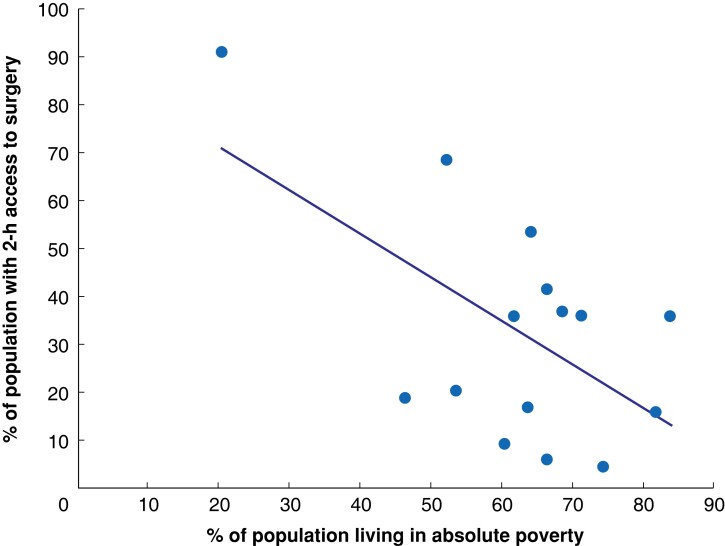
Population 2-h access to surgical facilities in each county in relation to county poverty rate Each data point represents 1 of the 15 counties in Liberia. *R*^2^ = 0.34.

Six facilities performed all bellwether procedures; 38.7 per cent of the population (1 751 000 people) had 2-h access to one of these facilities (*Fig. 2a*). Five facilities were identified as suitable candidates for scale up of bellwether capability (*Fig. 2b*) and efforts to strengthen these facilities could increase population access by 8.1 per cent (367 000 people). There were large disparities in surgical access between geographical areas (*Table 1*). Many surgical facilities did not meet any of the defined surgical standards (*Fig. 3*). People living in counties with high population poverty had the worst access to surgical care (*Fig. 4*).

**Table 1:** Proportion of population in each county with 2-h access to a facility meeting the defined surgical standards

	% of population				
	Any surgical facility	Safe surgery equipment	Specialist personnel	Bellwether capability	All three standards met
Montserrado (1 413 000)	90.8 (1 283 000)	90.7 (1 282 000)	90.4 (1 278 000)	89.7 (1 268 000)	89.4 (1 263 000)
Margibi (347 000)	68.3 (237 000)	62.5 (217 000)	69.5 (241 000)	61.1 (212 000)	61.1 (212 000)
Bomi (157 000)	52.9 (83 000)	46.5 (73 000)	42.0 (66 000)	41.4 (65 000)	35.7 (56 000)
Nimba (586 000)	41.3 (242 000)	37.9 (222 000)	12.6 (74 000)	27.3 (160 000)	0 (0)
Lofa (341 000)	36.7 (125 000)	25.8 (88 000)	0.9 (3000)	0 (0)	0 (0)
Bong (385 000)	35.8 (138 000)	35.6 (137 000)	30.4 (117 000)	9.9 (38 000)	0.3 (1000)
Maryland (187 000)	35.8 (67 000)	33.7 (63 000)	33.7 (63 000)	0 (0)	0 (0)
Grand Bassa (261 000)	35.6 (93 000)	32.2 (84 000)	2.7 (7000)	1.2 (3000)	1.2 (3000)
Grand Cape Mount (168 000)	20.2 (34 000)	11.9 (20 000)	1.2 (2000)	1.2 (2000)	0.6 (1000)
Sinoe (130 000)	18.5 (24 000)	0 (0)	0 (0)	0 (0)	0 (0)
Grand Gedeh (175 000)	17.1 (30 000)	3.4 (6000)	3.4 (6000)	0 (0)	0 (0)
River Gee (107 000)	15.9 (17 000)	0.5 (500)	0.5 (500)	− (0)	− (0)
Gbapolu (110 000)	9.1 (10 000)	4.6 (5000)	0.9 (1000)	0.9 (1000)	0.5 (500)
River Cess (98 000)	6.1 (6000)	1.0 (1000)	− (0)	− (0)	− (0)
Grand Kru (64 000)	4.7 (3000)	− (0)	− (0)	− (0)	− (0)
Nationally (4 525 000)	52.9 (2 392 000)	48.6 (2 199 000)	41.1 (1 858 000)	38.7 (1 751 000)	34.0 (1 538 000)

Values in parentheses are population numbers rounded to closest 1000 or 100.

### Performance of other essential surgical procedures

Apart from the bellwether procedures, a spectrum of 22 different essential surgical procedures were found in the operative logs from surgical facilities throughout Liberia (*Table S1*). Facilities with safe surgery equipment available performed a median of 6.0 (i.q.r. 3.0–9.0) other essential procedures, whereas those with specialist personnel present performed 11.5 (6.8–16.3) other essential procedures. Bellwether-capable facilities provided 13.0 (11.3–16.5) other essential procedures. Among facilities meeting all three standards, a median of 14.0 (11.5–15.0) other essential procedures were performed. Many other essential procedures were not performed by bellwether-capable facilities (*Table S2*).

### Surgical standards and their association with other essential procedures

No clear association was detected between availability of safe surgery equipment (OR 0.89, 95 per cent c.i. 0.48 to 1.67; *P* = 0.716) or presence of both types of specialist (OR 1.39, 0.71 to 2.72; *P* = 0.342) and performance of a broader range of essential surgical procedures (*Table 2*). Bellwether-capable facilities had a 3.13 (1.28 to 7.65) times higher odds of performing any of the other essential procedures compared with facilities that did not perform any bellwether procedures (*P* = 0.012).

**Table 2:** Association between surgical facility standards and performance of other essential procedures

	No. of surgical facilities	Univariable model		Multivariable model	
		OR	*P*	OR	*P*
**Safe surgery equipment**					
ȃNot available	24	1.00 (reference)		1.00 (reference)	
ȃAvailable	27	1.76 (1.00, 3.11)	0.049	0.89 (0.48, 1.67)	0.716
**Specialist present**					
ȃNo specialist	28	1.00 (reference)		1.00 (reference)	
ȃSurgeon	5	1.54 (0.69, 3.47)	0.292	2.09 (0.96, 4.53)	0.062
ȃObstetrician/gynaecologist	8	1.10 (0.56, 2.18)	0.787	0.60 (0.33, 1.09)	0.093
ȃBoth types of specialist	10	4.62 (2.53, 8.44)	<0.001	1.39 (0.71, 2.72)	0.342
**No. of bellwethers**					
ȃ0	6	1.00 (reference)		1.00 (reference)	
ȃ1	14	1.06 (0.48, 2.33)	0.882	0.92 (0.46, 1.81)	0.798
ȃ2	25	2.28 (1.11, 4.68)	0.025	1.00 (0.47, 2.09)	0.989
ȃAll	6	10.35 (4.33, 24.73)	<0.001	3.13 (1.28, 7.65)	0.012
All surgical standards*					
ȃNot meeting all standards	48	1.00 (reference)		1.00 (reference)	
ȃMeeting all 3 standards	3	5.23 (1.75, 15.62)	0.003	2.03 (0.91, 4.51)	0.082
**Volume of bellwethers (no. of procedures)†**					
ȃ0–3	12	1.00 (reference)		1.00 (reference)	
ȃ4–27	13	1.51 (0.75, 3.01)	0.245	1.28 (0.68, 2.44)	0.446
ȃ28–81	13	2.60 (1.33, 5.11)	0.005	1.15 (0.51, 2.57)	0.739
ȃ82–535	13	6.05 (3.11, 11.75)	<0.001	1.54 (0.60, 3.91)	0.370
**Caesarean section**					
ȃNot performing	7	1.00 (reference)		1.00 (reference)	
ȃPerforming	44	1.80 (0.77, 4.19)	0.176	0.71 (0.34, 1.47)	0.358
**Laparotomy**					
ȃNot performing	19	1.00 (reference)		1.00 (reference)	
ȃPerforming	32	3.33 (1.96, 5.65)	<0.001	1.36 (0.77, 2.39)	0.294
**Treatment of open fracture**					
ȃNot performing	45	1.00 (reference)		1.00 (reference)	
ȃPerforming	6	6.43 (3.25, 12.74)	< 0.001	3.16 (1.86, 5.38)	<0.001

Values in parentheses are 95% confidence intervals. *Availability of safe surgery equipment, presence of surgeon and obstetrician/gynaecologist, and performance of all three bellwether procedures.†Four-month volume (total number) of the three bellwether procedures.

## Discussion

This study found that half of the people in Liberia cannot reach their closest surgical facility, regardless of resources present, within 2 h of travel. About 60 per cent of the population does not have 2-h access to a bellwether-capable facility. Surgical facilities that performed the bellwether procedures were more likely to offer a broader range of other essential procedures; regardless, these facilities only delivered a median of 13 additional essential procedures.

Identifying strategies to reduce delays in reaching and receiving surgical care will be key if Liberia is to achieve the Lancet Commission on Global Surgery^1^ target of 80 per cent population 2-h access to essential surgical care. The country is struggling with poor road conditions hindering geographical access. In Grand Kru County, for example, reducing travel times is necessary as only 5 per cent of the county population can reach the closest surgical facility within 2 h of travel. In Lofa County, however, improving bellwether capability can be a reasonable approach as 37 per cent of the population already has geographical access, but essential services are largely unavailable for those who can reach the facilities. This analysis offers guidance for local policy priorities and presents a baseline for future geospatial analysis to be measured against.

Surgical facilities performing the bellwether procedures are thought to function ‘at a level of complexity advanced enough to do most other surgical procedures’^1^. The present analysis confirms that bellwether-capable facilities are more likely to perform other essential procedures. Nevertheless, bellwether-capable facilities only performed a median of 13 additional essential procedures. The third edition of the Disease Control Priorities^2^ lists 22 essential procedures other than the bellwethers that should be performed in all first-level hospitals. A survey of 905 facilities from the WHO Emergency and Essential Surgical Care Global database found a statistically significant correlation between bellwether capability and 24 other essential procedures^3^. Hence, bellwether-capable facilities in Liberia also fell short of providing the most basic range of essential surgical care. It is therefore questionable whether bellwether capability is a valid proxy for the full breadth of essential surgery, especially in low-income countries like Liberia where surgical providers are sparse^13^ and surgical volumes are low^12^.

To draw a clear line between facilities in which essential services are available and those where such services are lacking is challenging yet necessary if the 2-h access indicator is to be useful. The bellwether procedures represent a practical threshold, but lack of definitional clarity hampers their comparability and utility^23^. Recently, an international Delphi exercise defined a globally applicable ‘basket’ of 32 surgical procedures with the purpose of standardizing surgical system assessments across countries and over time^7^. Applying a more comprehensive list of well defined surgical procedures to identify facilities capable of delivering essential surgical care is likely to improve the comparability and validity of the 2-h access indicator. Such a benchmark can also identify services that might be lacking and in need of resourcing.

To deliver an adequate volume and range of essential procedures, a well equipped surgical environment with trained personnel is necessary. The number of surgical personnel and infrastructure have previously been shown to correlate with operative output in Liberia^12^. However, in the present study, the presence of specialists and safe surgery equipment did not translate into a wide range of essential procedures. Furthermore, a combination of specialist, safe surgery equipment, and bellwether capability standards were less likely to predict surgical breadth than the bellwether standard alone. Infrastructure and workforce are essential building blocks of any healthcare system, and the WHO recommends routine surveillance of these standards^24^. It is, however, important for policymakers to keep in mind that the presence of these standards may not always reflect adequate output of surgical procedures.

Some limitations should be considered when interpreting the study results. First, the analysis did not account for the delay in seeking care. Second, the present geospatial model did not adjust for fluctuating road conditions due to changes in weather and traffic, which are variables known to influence travel times^25,26^. Third, the analysis did not consider affordability aspects, which are known to influence people’s ability to access surgical care^27^. Fourth, the ability to deliver surgical procedures consistently over time and the quality of the services were not assessed. Finally, some of the specialized essential procedures may have been missed. Surgical missions performing cleft lip and palate surgery might have been missed by the 4-month sample. Furthermore, cataract surgery is being performed in the main teaching hospital in Monrovia, but this was missed during the data collection because the eye clinic is in a separate building away from the other operating theatres.

Population access to essential surgical services in Liberia is limited. Some areas, such as Grand Kru County, need to reduce travel times owing to poor geographical access. Other areas, like Lofa County, have somewhat better geographical access, but lack facility resources to deliver essential surgical care. People who live in more urban areas, such as Montserrado Country where the capital Monrovia is located, have better access to essential surgical services, and there is limited room for improvement. The validity of these estimates, however, relies on a relevant definition of capable. Although bellwether-capable facilities performed a broader range of other essential surgical procedures, they did not deliver the full breadth of essential surgical care. Hence, bellwether capability as a proxy for the delivery of most other essential procedures may not be equally valid across all types of setting. To improve the validity of the 2-h access indicator, it may be necessary to look beyond the bellwethers and define a broader sample of surgical procedures that fully reflects the delivery of essential surgical care.

## Supplementary Material

znac377_Supplementary_Data

## Data Availability

The data material for this study will be made available upon reasonable request.
