# Supplementary material for: Mapping population access to essential surgical care in Liberia using equipment, personnel, and bellwether capability standards
Source: Br J Surg. 2022 Dec 5;110(2):169–76. doi: 10.1093/bjs/znac377 (PMC10364551; doi:10.1093/bjs/znac377)
Supplement: znac377_Supplementary_Data [file znac377_supplementary_data.docx]

**Mapping population access to essential surgical care in Liberia using equipment, personnel, and Bellwether capability standards**

Håvard A. Adde^1^, Alex J. van Duinen^1,2^, Benetta C. Andrews^3^, Juul Bakker^1^, Kezelebah S. Goyah^4,5^, Øyvind Salvesen^6^, Swaliho Sheriff^4,7^, Terseer Utam^4,8^, Clarence Yaskey^4^, Thomas G. Weiser^9,10,11,12^, Håkon A. Bolkan^2,6^

1. Department of Clinical and Molecular Medicine, Faculty of Medicine and Health Sciences, NTNU –Norwegian University of Science and Technology, Trondheim, Norway.
2. Department of Surgery, St Olav’s Hospital, Trondheim University Hospital, Trondheim, Norway.
3. Liberia College of Physicians and Surgeons, Monrovia, Liberia.
4. Lifebox Foundation, Monrovia, Liberia.
5. F. J. Grante Memorial Hospital, Greenville, Liberia.
6. Department of Public Health and Nursing, Faculty of Medicine and Health Sciences, NTNU –Norwegian University of Science and Technology, Trondheim, Norway.
7. Department of Surgery, Liberia Governmental Hospital, Tubmanburg, Liberia.
8. Department of Surgery and Traumatology, Redemption Hospital, Monrovia, Liberia.
9. Department of Surgery, Stanford University, Stanford, USA.
10. Stanford-Surgery Policy Improvement Research and Education Center, Department of Surgery, Stanford University, Palo Alto, USA.
11. Department of Clinical Surgery, University of Edinburgh, Edinburgh, UK.
12. Lifebox Foundation, London, UK.

**Corresponding author:**

Håvard Askim Adde, MD
NTNU – Norwegian University of Science and Technology
Department of Clinical and Molecular Medicine

Post Box 8905, NO-7491 Trondheim, Norway

Email: haavarad@stud.ntnu.no

Phone: +47 48145076

ORCID: 0000-0002-0883-0549

Twitter: @AskimAdde

**Supplementary Materials - Index**

| **Supplementary Figures and Tables** |  |
| --- | --- |
| Table S1 | *pag. 3* |
| Table S2 | *pag. 4* |

**Supplementary Figures and Tables**

Table S1: Essential surgical procedures other than the Bellwethers (caesarean section, laparotomy, and open fracture treatment) performed throughout Liberia.

| **Other essential surgical**  **procedures performed** | **Other essential surgical procedures not performed** |  |
| --- | --- | --- |
| Salpingectomy for ectopic pregnancy | Vasectomy | Obstetrics/gynaecology |
| Hysterectomy | Cryotherapy for precancerous cervical lesions |  |
| Dilatation & curettage | Repair obstetric fistula |  |
| Tubal ligation |  |  |
| Hernia repair |  | General surgery |
| Appendectomy |  |  |
| Drainage of abscess |  |  |
| Colostomy |  |  |
| Cholecystectomy |  |  |
| Circumcision male |  |  |
| Hydrocelectomy |  |  |
| Catheterization or suprapubic cystostomy |  |  |
| Urethral stricture dilatation |  |  |
| Osteomyelitis debridement | Surgical airway | Injury/orthopaedics |
| Skin grafting | Burr hole |  |
| Escharotomy/fasciotomy | Drainage of septic arthritis |  |
| Suturing lacerations |  |  |
| Wound debridement |  |  |
| Amputation |  |  |
| Chest tube |  |  |
| Repair of club foot | Repair of anorectal malformation and Hirschsprung’s disease | Congenital |
| Shunt for hydrocephalus | Cleft lip and palate repair |  |
|  | Cataract extraction and insertion of intraocular lens | Visual impairment |
|  | Eyelid surgery for trachoma |  |

Table S2: Four-month volume (total number) of other essential surgical procedures performed among the six Bellwether capable facilities.

|  | **Bellwether facility** | | | | | |
| --- | --- | --- | --- | --- | --- | --- |
|  | **A** | **B** | **C** | **D** | **E** | **F** |
| Repair of club foot | 0 | 0 | 1 | 0 | 0 | 0 |
| Dilatation for urethral stricture | 0 | 0 | 4 | 0 | 0 | 1 |
| Catheterization/suprapubic puncture | 3 | 0 | 4 | 1 | 1 | 0 |
| Tubal ligation | 0 | 0 | 0 | 0 | 0 | 0 |
| Skingraft | 0 | 0 | 9 | 1 | 2 | 6 |
| Shunt for hydrocephalus | 0 | 0 | 0 | 0 | 1 | 0 |
| Scrotal hydrocele | 0 | 3 | 1 | 1 | 2 | 0 |
| Salpingectomy for ectopic pregnancy | 13 | 0 | 11 | 7 | 14 | 2 |
| Osteomyelitis debridement | 5 | 1 | 6 | 1 | 17 | 2 |
| Wound debridement | 5 | 2 | 24 | 0 | 14 | 8 |
| Drainage of abscess | 6 | 2 | 4 | 7 | 13 | 0 |
| Hysterectomy | 4 | 1 | 5 | 4 | 28 | 1 |
| Hernia repair | 30 | 8 | 52 | 29 | 81 | 11 |
| Escharotomy | 0 | 0 | 0 | 0 | 1 | 0 |
| Dilatation and curettage | 54 | 0 | 36 | 24 | 4 | 2 |
| Colostomy | 0 | 0 | 5 | 1 | 4 | 0 |
| Circumcision male | 0 | 0 | 1 | 0 | 0 | 1 |
| Cholecystectomy | 0 | 0 | 0 | 0 | 0 | 0 |
| Chest tube | 4 | 0 | 2 | 2 | 0 | 3 |
| Appendectomy | 6 | 2 | 9 | 12 | 15 | 0 |
| Wound suturing | 15 | 4 | 15 | 7 | 7 | 1 |
| Amputation | 5 | 1 | 14 | 3 | 6 | 4 |
